# IgA nephropathy is associated with elevated urinary mitochondrial DNA copy numbers

**DOI:** 10.1038/s41598-019-52535-5

**Published:** 2019-11-05

**Authors:** Byung Chul Yu, Nam-Jun Cho, Samel Park, Hyoungnae Kim, Soo Jeong Choi, Jin Kuk Kim, Seung Duk Hwang, Hyo-Wook Gil, Eun Young Lee, Jin Seok Jeon, Hyunjin Noh, Dong Cheol Han, Yon Hee Kim, So-Young Jin, Moo Yong Park, Soon Hyo Kwon

**Affiliations:** 1 Division of Nephrology, Department of Internal Medicine, Soonchunhyang University Bucheon Hospital, 170 Jomaru-ro, Bucheon, 14584 Republic of Korea; 2 Division of Nephrology, Department of Internal Medicine, Soonchunhyang University Cheonan Hospital, 31, Suncheonhyang 6-gil, Dongnam-gu, Cheonan, 31151 Republic of Korea; 3 Division of Nephrology, Department of Internal Medicine, Soonchunhyang University Seoul Hospital, 59, Daesagwan-ro, Yongsan-gu, Seoul, 04401 Republic of Korea; 4 Department of Pathology, Soonchunhyang University Seoul Hospital, 59, Daesagwan-ro, Yongsan-gu, Seoul, 04401 Republic of Korea

**Keywords:** Predictive markers, Prognostic markers, IgA nephropathy, Glomerulus

## Abstract

Mitochondrial injury plays important roles in the pathogenesis of various kidney diseases. However, mitochondrial injury in IgA nephropathy (IgAN) remains largely unexplored. Here, we examined the associations among mitochondrial injury, IgAN, and treatment outcomes. We prospectively enrolled patients with IgAN and age-/sex-matched healthy volunteers (HVs) as controls (*n* = 31 each). Urinary copy numbers of the mitochondrial DNA (mtDNA) genes cytochrome-c oxidase-3 (*COX3*) and nicotinamide adenine dinucleotide dehydrogenase subunit-1 (*ND1*) were measured. Urinary mtDNA levels were elevated in the IgAN group compared with that in HVs (*p* < 0.001). Urinary *ND1* levels were significantly higher in the low proteinuria group than in the high proteinuria group (*p* = 0.027). Changes in urinary levels of *ND1* and *COX3* were positively correlated with changes in proteinuria (*p* = 0.038 and 0.024, respectively) and inversely correlated with changes in the estimated glomerular filtration rate (*p* = 0.033 and 0.017, respectively) after medical treatment. Mitochondrial injury played important roles in IgAN pathogenesis and may be involved in early-stage glomerular inflammation, prior to pathological changes and increased proteinuria. The correlation between changes in urinary mtDNA and proteinuria suggest that these factors may be promising biomarkers for treatment outcomes in IgAN.

## Introduction

IgA nephropathy (IgAN) is a type of mesangial proliferative glomerulonephritis (GN) characterized by diffuse deposition of IgA in the glomerular mesangium^1^ and is the most common cause of primary GN worldwide^2^ since its first description in 1968^3^. Within 20 years after diagnosis, one-fourth of patients will progress to end-stage renal disease (ESRD), and an additional 20% will have a gradual decline in renal function.

Although the data are not always consistent, hypertension (HTN), proteinuria, reduced glomerular filtration rate (GFR), and unfavorable histopathological findings at the time of diagnosis have all been shown to be associated with poor prognosis in IgAN^1^. Renal biopsy is indispensable for diagnosis and prediction of prognosis^4^. However, because renal biopsy is an invasive diagnostic method and cannot be performed periodically, there are limitations to validating treatment outcomes and predicting the prognoses. Moreover, there is a growing interest in identification of biomarkers as a method to complement the limitations of renal biopsy^5^.

The kidney has high energy demand and is rich in mitochondria. Moreover, mitochondrial dysfunction plays an important role in the pathogenesis of various kidney diseases, including focal segmental glomerulosclerosis^6,7^, acute kidney injury (AKI)^8^, chronic kidney disease (CKD)^9,10^, and obesity-related hyperfiltration^11^. Data are inadequate regarding the relationship between IgAN and mitochondrial injury. Cases of patient with mitochondrial disease and presence of a mutation in the mitochondrial gene accompanied by IgAN were reported^12,13^. Mitochondrial genetic variants in patients with IgAN and ESRD were investigated in a previous study^14^. Acquired factors associated with kidney diseases, such as oxidative stress^9,15^, and ischemia/hypoxia^16^, renin-angiotensin-aldosterone system activation^17,18^, proteinuria^19–21^, cause mitochondrial dysfunction. Mitochondrial dysfunction induces podocyte injury, tubular cell damage, and endothelial cell damage^22^. When mitochondrial injury occurs, fragments of mitochondrial DNA (mtDNA) are released into the cytosol from the matrix and then enter into the systemic circulation^23,24^. In addition, mitochondrial injury in the kidney may result in the release of these fragments into the urine. Previous studies have shown that urinary mtDNA copy numbers can be considered surrogate markers of mitochondrial injury in various kidney diseases^25–27^.

Accordingly, we hypothesized that the pathogenesis of IgAN may be associated with mitochondrial injury and that urinary mtDNA may serve as a valuable biomarker for evaluating treatment outcomes and predicting prognosis in patients with IgAN. In this study, we examined whether urinary mtDNA was elevated in patients with IgAN and assessed correlation with treatment outcomes and existing prognostic markers.

## Results

### Baseline characteristics of the patients and healthy volunteers (HVs)

Table 1 shows baseline characteristics of patients in the IgAN group and matched HVs. The mean serum creatinine (SCr) level and body mass index were higher, and the estimated GFR (eGFR) was lower in the IgAN group than in HVs. The proteinuria levels and systolic blood pressure (SBP) were significantly higher in the IgAN group than in HVs. The median value of proteinuria in the IgAN group was 1508.5 mg/day. We divided the IgAN group into high and low proteinuria groups based on a cut-off of 1500 mg/day.

**Table 1:** Baseline characteristics of both groups.

Variables	IgAN (*n* = 31)	HV (*n* = 31)	*p*-value
Sex (Male), *n* (%)	17 (54.8)	17 (54.8)	>0.999
Age (years)	40.55 ± 13.69	35.07 ± 10.71	0.084
Body mass index (kg/m^2^)	25.90 ± 4.99	22.39 ± 1.40	0.001
SCr (mg/dL)	1.24 ± 0.54	0.83 ± 0.15	<0.001
eGFR (mL/min/1.73 m^2^)	74.30 ± 28.72	107.21 ± 11.89	<0.001
Proteinuria (mg/24 h)	1848.2 ± 1559.6	65.11 ± 20.75	<0.001
SBP (mmHg)	132.9 ± 19.7	109.7 ± 9.1	<0.001
DBP (mmHg)	80.1 ± 15.3	74.1 ± 7.8	0.054
Mean arterial pressure (mmHg)	97.7 ± 16.0	85.9 ± 7.3	0.001
Oxford classification, *n* (%)			
M score 1	21 (67.7)	n.d.	
E score 1	13 (41.9)	n.d.	
S score 1	21 (67.7)	n.d.	
T score 1	14 (45.2)	n.d.	
T score 2	2 (6.5)	n.d.	
C score 1	8 (25.8)	n.d.	
C score 2	2 (6.5)	n.d.	
Log_10_*ND1*/nDNA (copies/μL urine/nDNA)	5.628 ± 0.404	5.158 ± 0.357	<0.001
Log_10_*COX3*/nDNA (copies/μL urine/nDNA)	5.619 ± 0.404	5.171 ± 0.336	<0.001
KIM-1 (ng/mL urine)	3.893 ± 3.946	0.577 ± 1.352	<0.001

Data are shown as mean ± standard deviation for continuous variables or n (%) for categorical variables. SCr: serum creatinine, eGFR: estimated glomerular filtration rate, SBP: systolic blood pressure, DBP: diastolic blood pressure, *ND1*: nicotinamide adenine dinucleotide dehydrogenase subunit-1, *COX3*: cytochrome-c oxidase-3, KIM-1: kidney injury molecule-1, IgAN: IgA nephropathy, HV: healthy volunteer, n.d.: not determined.

### Urinary mtDNA was elevated in patients with IgAN

**Figure 1:**
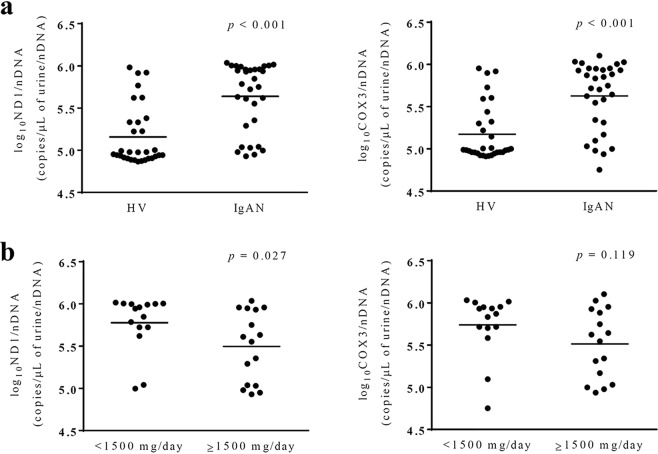
Urinary mitochondrial DNA (mtDNA) copy numbers at baseline. (**a**) Urinary mtDNA copy numbers were elevated in the IgA nephropathy (IgAN) group compared with those in healthy volunteers (HVs). (**b**) Urinary copy numbers of nicotinamide adenine dinucleotide dehydrogenase subunit-1 (*ND1*) were higher in the low proteinuria group than in the high proteinuria group. Data were analyzed by Mann-Whitney tests. *COX3*: cytochrome-c oxidase-3.

Log_10_ nicotinamide adenine dinucleotide dehydrogenase subunit-1 (*ND1*)/nDNA and log_10_ cytochrome c oxidase subunit 3 (*COX3*)/nDNA were elevated in the IgAN group compared with those in HVs (Fig. 1a). Urinary *ND1* levels were significantly higher in the low proteinuria group than in the high proteinuria group (Fig. 1b). Urinary *ND1* and *COX3* levels were significantly higher in both subgroups compared with those in HVs.

### Urinary mtDNA did not correlate with the existing prognostic markers at presentation of IgAN

There were no significant relationships between urinary mtDNA copy numbers and traditional prognostic markers, including mean arterial pressure, eGFR, and baseline proteinuria levels, at presentation in the IgAN group (Table 2). Urinary *ND1* and *COX3* did not correlate with the MEST-C score of the Oxford classification, but tended to be higher in patients with M0 and T0 (Table 3).

**Table 2:** Relationship between urinary mitochondrial DNA levels and clinical variables in patients with IgA nephropathy.

Variables	Mean arterial pressure	Estimated glomerular filtration rate	Amount of proteinuria
Log_10_*ND1*/nDNA level	r = −0.166, *p* = 0.372	r = 0.279, *p* = 0.128	r = −0.258, *p* = 0.128
Log_10_*COX3*/nDNA level	r = −0.127, *p* = 0.495	r = 0.199, *p* = 0.283	r = −0.119, *p* = 0.523

Data were analyzed by Spearman’s rank correlation coefficient. *ND1*: nicotinamide adenine dinucleotide dehydrogenase subunit-1, *COX3*: cytochrome-c oxidase-3.

**Table 3:** Relationship between urinary mitochondrial DNA levels and Oxford classification in patients with IgA nephropathy.

Variables	Log_10_*ND1*/nDNA (copies/μL urine/nDNA)	*p*-value	Log_10_*COX3*/nDNA (copies/μL urine/nDNA)	*p*-value
Oxford classification				
M score 0	5.756 ± 0.414	0.079	5.734 ± 0.380	0.227
1	5.562 ± 0.381		5.560 ± 0.403	
E score 0	5.615 ± 0.433	0.704	5.609 ± 0.446	0.765
1	5.658 ± 0.350		5.643 ± 0.324	
S score 0	5.571 ± 0.435	0.670	5.568 ± 0.447	0.699
1	5.664 ± 0.383		5.652 ± 0.376	
T score 0	5.654 ± 0.426	0.401	5.649 ± 0.397	0.800
1, 2	5.607 ± 0.378		5.593 ± 0.410	
C score 0	5.578 ± 0.415	0.428	5.573 ± 0.413	0.273
1, 2	5.763 ± 0.339		5.741 ± 0.350	

Data are shown as mean ± standard deviation for continuous variables and were analyzed by Mann-Whitney *U*-tests. *ND1*: nicotinamide adenine dinucleotide dehydrogenase subunit-1, *COX3*: cytochrome-c oxidase-3.

### Urinary mtDNA did not correlate with renal injury markers

**Figure 2:**
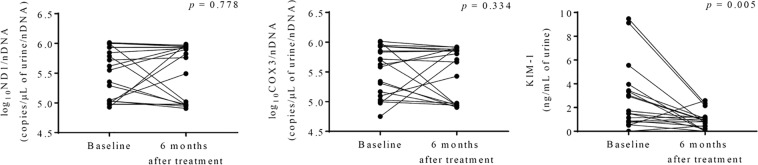
Changes in urinary mitochondrial DNA copy numbers and kidney injury molecule-1 (KIM-1) levels at 6 months after medical treatment. Data were analyzed by Wilcoxon matched-pairs signed rank tests. *COX3*: cytochrome-c oxidase-3, *ND1*: nicotinamide adenine dinucleotide dehydrogenase subunit-1.

**Figure 3:**
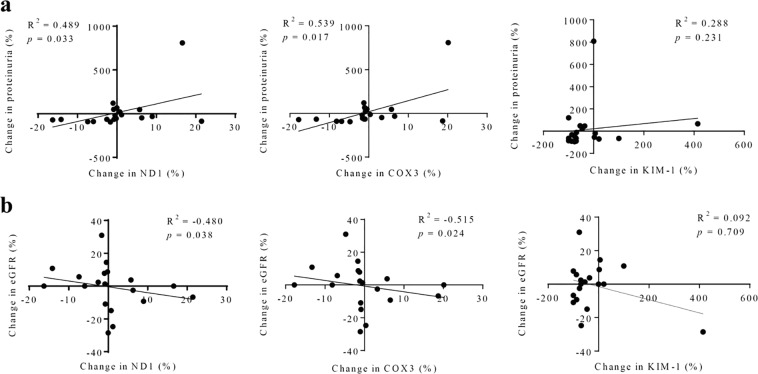
Relationships among changes in urinary mitochondrial DNA (mtDNA), kidney injury molecule-1 (KIM-1), proteinuria, and estimated glomerular filtration rate (eGFR) after medical treatment. Changes in urinary levels of mtDNA showed positive correlations with changes in proteinuria at 6 months (**a**) and were inversely correlated with changes in eGFR at 12 months after medical treatment. (**b**) Data were analyzed by Spearman’s rank correlation coefficient. *ND1*: nicotinamide adenine dinucleotide dehydrogenase subunit-1, *COX3*: cytochrome-c oxidase-3.

Urinary kidney injury molecule-1 (KIM-1) level were significantly higher in the IgAN group than in HVs (Table 1). Urinary *ND1* (*r* = −0.087, *p* = 0.643) and *COX3* (*r* = −0.022, *p* = 0.905) levels did not correlate with urinary KIM-1 levels. Changes in *ND1* (*r* = −0.125, *p* = 0.611) and *COX3* (*r* = −0.246, *p* = 0.310) urinary levels also did not correlate with changes in KIM-1 levels 6 months after medical treatment. Urinary KIM-1 levels were positively correlated with baseline proteinuria levels (*r* = 0.556, *p* = 0.001) and were related to endocapillary proliferation according to the Oxford classification (E0, 2.306 ± 2.310; E1, 6.405 ± 4.743 ng/mL urine; *p* = 0.008). KIM-1 levels were significantly reduced after medical treatement (*p* = 0.005; Fig. 2). Changes in KIM-1 levels did not correlate with changes in proteinuria or eGFR at 6 and 12 months after medical treatment. However, changes in KIM-1 levels tended to be positively and inversely correlated with changes in proteinuria and eGFR, respectively (Fig. 3).

### Treatment did not affect the levels of urinary mtDNA in patients with IgAN

Urinary mtDNA in 19 patients in the IgAN group was measured 6 months after medical treatment. In followed patients, urinary *ND1* and *COX3* were higher in the high proteinuria group than in the low proteinuria group (*p* = 0.007 and 0.016, respectively). All 19 patients were treated with angiotensin receptor blockers (ARBs), and six were treated with an immunosuppressive agent (five patients were treated with corticosteroids, and one patient was treated with cyclosporin). Medical treatment did not reduce urinary levels of *ND1* and *COX3* (*p* = 0.778 and 0.334, respectively). Baseline proteinuria levels were lower (*p* = 0.029) and urinary *ND1* and *COX3* levels were higher (*p* < 0.001, both) in patients treated only with ARBs (only ARB treatment group) than those treated with ARBs and immunosuppressant (concurrent treatment group) in IgAN patients. Urinary levels of *ND1* and *COX3* were not reduced 6 month after treatment in concurrent treatment group (*p* = 0.917, both) and only ARBs treatment group (*p* = 0.701, 0.221, respectively).

**Figure 4:**
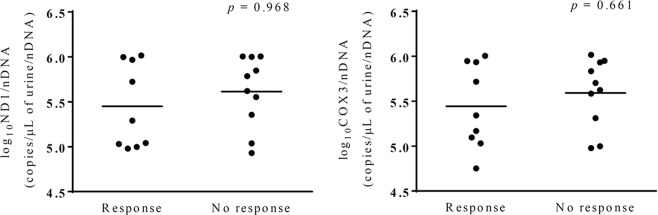
Relationship between baseline urinary mitochondrial DNA copy number and treatment response. Data were analyzed by Mann-Whitney tests. *ND1*: nicotinamide adenine dinucleotide dehydrogenase subunit-1, *COX3*: cytochrome-c oxidase-3.

Changes in proteinuria and eGFR after treatment correlated with changes in urinary mtDNA copy numbers in patients with IgAN. Moreover, changes in urinary levels of *ND1* and *COX3* showed positive correlations with changes in proteinuria (*p* = 0.038, 0.024, respectively) at 6 months and were inversely correlated with changes in eGFR at 12 months (Fig. 3) after medical treatment. There were no significant differences in baseline urinary *ND1* (*p* = 0.968) and *COX3* (*p* = 0.661) levels between the response and no response groups (Fig. 4).

### Structural changes in mitochondria were detected in the IgAN group

**Figure 5:**
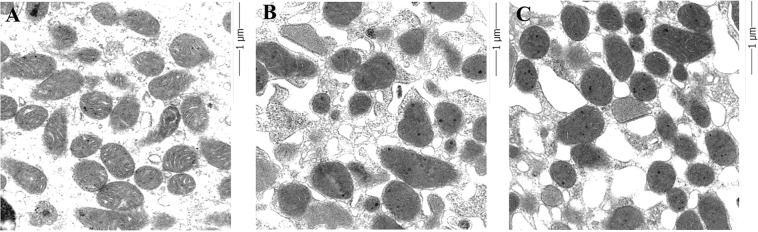
Comparison of mitochondria from living kidney donors who had normal kidney function and patients with IgA nephropathy (IgAN). (**A**) Representative electron microscopy (EM) image of proximal tubular (PT) epithelial cells from a living kidney donor who had a normal kidney structure. Many elongated mitochondria harbored densely stacked cristae membranes that were organized along membrane compartments. (**B**,**C**) Representative EM images of PT epithelial cells in patients with IgAN. Mitochondria were small and disorganized. Cristae membranes were replaced by the homogenized matrix. Electron-dense particles or droplets are shown in a few mitochondria.

Electron microscopic images showed that the mitochondria in the tubular epithelium and glomerular endothelial cells in kidney donors had many elongated mitochondria with densely stacked cristae membranes that were organized along membrane compartments. However, mitochondria were small and disorganized in patients with IgAN. Cristae membranes from some patients were replaced by a homogenized matrix, and electron-dense particles or droplets were detected in a few mitochondria (Fig. 5). These results suggested that structural changes occurred in the mitochondria of patients with IgAN.

## Discussion

In this study, we evaluated changes in urinary mtDNA in patients with IgAN. Our results showed that urinary *ND1* and *COX3* copy numbers were well correlated with each other and were higher in the IgAN group than in HVs. This result suggested that mitochondrial injury was associated with IgAN. During the follow-up period, changes in urinary mtDNA correlated with changes in eGFR and proteinuria. Because changes in albuminuria are an independent prognostic marker in IgAN^28,29^, urinary mtDNA could be a promising biomarker for IgAN.

We also analyzed correlations between urinary mtDNA and existing clinical and histopathological prognostic markers at presentation in IgAN and found that there were no correlations. These findings are not consistent with previous studies in AKI, HTN, and diabetes mellitus (DM) nephropathy^25–27^. Urinary mtDNA levels tended to be inversely correlated with baseline proteinuria levels and were higher in patients who had a mesangial score of less than 0.5 or tubular atrophy/interstitial fibrosis less than or equal to 25% (Oxford classification M0 or T0, respectively). Interestingly, when dividing the IgAN group into two subgroups according to baseline proteinuria, a subgroup of patients with lower proteinuria showed higher urinary mtDNA levels. In the same context, because of more patients with high proteinuria were treated with immunosuppressant, only ARB treatment group showed lower baseline proteinuria levels compared with concurrent treatment group. However, baseline urinary mtDNA levels were higher in only ARB treatment group than concurrent treatment group.

According to our current knowledge, the immunopathogenesis of IgAN involves a multi-hit pathogenic process. The presence of increased amounts of underglycosylated IgA1 induces autoantibodies and the formation of immune complexes, which activate the complement pathway and contribute to mesangial activation and proliferation. Glomerular inflammation results in podocyte injury, proteinuria, tubulointerstitial inflammation, and fibrosis^30,31^. In our patients with IgAN, we found that urinary mtDNA copy numbers were higher in patients with less histological damage and proteinuria. These results may indicate that mitochondrial damage is involved in the early stage of glomerular inflammation and occurs prior to pathological changes. Accordingly, increased proteinuria and reduced renal function and urinary mtDNA may be the earliest signs of kidney injury. However, the duration of our study was relatively short; thus, further studies are needed to confirm these findings as potential prognostic markers in patients with IgAN.

Medical treatment for IgAN did not affect mtDNA copy numbers. Notably, body weight reduction after bariatric surgery did not decrease urinary mtDNA copy numbers in a previous study^11^. In contrast, KIM-1 levels decreased after medical treatment in our IgAN cohort, consistent with previous findings^32^. KIM-1 is a marker of tubule injury in the kidney; however, urinary mtDNA could have been derived from all cells in the nephron and glomerulus. These different sources could explain the lack of changes in mtDNA copy numbers in IgAN. Alternatively, urinary mtDNA copy numbers may reflect overall kidney cell damage and could be used as a unique renal injury marker distinct from existing renal injury markers.

Changes in urinary mtDNA levels were correlated with changes in eGFR and proteinuria levels in our study. Substantial observational data have supported that reduced proteinuria is associated with improved renal survival^31^. Moreover, a recent meta-analysis also showed that reduced proteinuria is associated with kidney outcomes, including doubling of SCr, ESRD, or death, and could be used as a surrogate endpoint in trials of IgAN^33^. Accordingly, it seems reasonable to conclude that reduced proteinuria is associated with improved kidney outcomes. In our study, percent reduction in urinary mtDNA copy numbers was found to be related to improved eGFR and decreased proteinuria. These findings suggest that urinary mtDNA copy numbers could also be used as a surrogate marker of kidney outcomes in IgAN. Taken together, these results suggest that alleviation of mitochondrial injury through preventive and therapeutic measures may improve clinical outcomes in IgAN, particularly at an early stage.

*COX3* and *ND1* encode the enzyme responsible for the mitochondrial respiratory chain and the electron transport, respectively. Previous studies showed that *ND1* did not differ from *COX3* copy numbers in obese African American hypertensive patients^34^ and upon chronic renal injury in hypertensive patients^25^. However, urinary *COX3* copy numbers from obese patients with glomerular hyperfiltration were not elevated compared with HVs^11^. In this study, urinary *ND-1*, but not *COX3* levels were higher in the low proteinuria group compared with the high proteinuria group. There are various factors that cause mitochondrial injury in kidney disease. We think the causes of mitochondrial injury may be different for each kidney disease, leading to differences in mtDNA injury patterns. Although there was no statistical significance, urinary *COX3* levels tended to be higher in the low- vs. high-proteinuria, and given the small sample size, *ND-1* is unlikely to be a better mitochondrial injury marker than *COX3*.

To our knowledge, there has been no study evaluating the urinary mtDNA levels in GN. Since we have evaluated only patients with IgAN, it is not clear whether elevated urinary mtDNA levels is a ubiquitous finding in GN or a unique finding in IgAN. To clarify this, future studies are needed to measure and compare the urinary mtDNA in each GN.

There were some limitations to our study. First, the sample size was small, and the duration of follow-up was short, which may have made the study underpowered for evaluating whether differences in urinary mtDNA levels were useful for predicting treatment responses or prognosis. Attenuated urinary mtDNA levels were correlated with decreased proteinuria and improved eGFR in our study; however, these findings should be confirmed further in additional studies with larger sample sizes and longer follow-up durations. Second, we did not measure serum mtDNA copy numbers. We measured urinary mtDNA levels, because previous studies showed that, unlike circulating mtDNA levels, urinary mtDNA levels are correlated with renal dysfunction and clinical outcomes in various kidney disease including AKI^35,36^, CKD^25^, and obesity-related hyperfiltration^11^. Age-/sex-matched HVs were collected to minimize the effects of other factors that could increase circulating mtDNA levels. However, patients with IgAN tended to be older and have higher body mass index than HVs. An important limitation of this study is that we could not measure circulating mtDNA levels, due to which we cannot exclude the possibility that urinary mtDNA levels were elevated due to increased circulating mtDNA levels by other factors in the urine. In addition, although urinary mtDNA was increased in patients with IgAN compared with HVs, the variation in IgAN group was so high that it was difficult to detect individual differences among patients. This limitation could be compensated by measuring circulating mtDNA. Finally, we could not explain the specific mechanisms through which mitochondrial injury leads to kidney damage or determine in which part of the kidney mitochondrial injury may occur. In early stage of IgAN pathogenesis, galactose-deficient IgA1-containing immune complexes activate mesangial cells that secrete components of extracellular matrix, releasing various mediators including proinflammatory cytokines^37–39^ and activating renin-angiotensin-alodosterone system^40–42^. These events may eventually lead to oxidative stress^43^ and mitochondrial dysfunction. Further studies are required to support this hypothesis. In this study, we identified ultrastructural changes in the mitochondria through kidney biopsy specimens of two patients with IgAN. However, we could not quantitatively measure the extent of damage and determine in which part of the kidney mitochondrial injury may occur. In future studies, if ultrastructural changes in the mitochondria of kidney biopsy specimen from patients with IgAN is measured quantitatively using electron microscope and compared with urinary mtDNA level, more accurate mitochondrial injury site could be determined.

In conclusion, our findings suggested that mitochondrial injury may play a role in the pathogenesis of IgAN and may be involved in early-stage glomerular inflammation prior to pathological changes and increased proteinuria. Mitochondrial injury may be associated with changes in proteinuria and eGFR and may therefore represent a promising biomarker for treatment outcomes in patients with IgAN.

## Methods

### Study population

This was a multicenter prospective cohort study. The current study was conducted according to the principles outlined in the Declaration of Helsinki, and clinical data from patients were obtained after approval of the study by the Institutional Review Board (IRB No. 2016-01-002-007) of Soonchunhyang University Hospital. We enrolled patients with biopsy-proven pure IgAN (*n* = 31) who were collected from the Cohort for Biomarker Inquiry of Renal Aggravation (COBRA) cohort at Soonchunhyang University Seoul, Bucheon, and Cheonan Hospitals from May 2016 to January 2018. Age- and sex-matched HVs were enrolled as controls (*n* = 31). HVs were included if they had no history of DM, HTN, congestive heart failure, coronary artery disease, and stroke and did not take any medications. All participants provided written informed consent.

### Clinical, laboratory, and pathologic data

We obtained data on demographics and comorbidities, including history of DM, HTN, hyperlipidemia, and hepatitis. We collected body weights, heights, SBP, and diastolic blood pressure at the time of kidney biopsy. We obtained information regarding the type, duration, and total dose of administered medications, including immunosuppressants and ARBs during the follow-up period. We collected laboratory data at every visit during follow-up. We determined the eGFR from SCr values using the CKD Epidemiology Collaboration equation. Proteinuria levels were determined by 24-h urine collection. We reviewed the pathological findings from kidney biopsies and specifically checked the pathological severity in patients with IgAN according to the Oxford classification^44^.

The IgAN group was classified into the low and high proteinuria groups according to baseline proteinuria levels based on median values. We subclassified patients with IgAN into the response group, defined as proteinuria less than 0.3 g/24 h, and the no response group, defined as proteinuria greater than or equal to 0.3 g/24 h or not decreased from the initial value at 6 months after medical treatment^45^.

### Urinary mtDNA levels

Urinary copy numbers of the mtDNA genes *COX3* and *ND1* were measured by quantitative real-time reverse transcription polymerase chain reaction (RT-PCR). We also measured urinary *ND1* and *COX3* levels 6 months after medical treatment in IgAN group (*n* = 19). DNA was isolated from urine samples (1.75 mL) using DNA isolation kits from Norgen Biotek (Thorold, ON, Canada; cat. no. 18100). We analyzed DNA concentrations using a NanoDrop Spectrophotometer (Thermo Fisher Scientific, Waltham, MA, USA). RT-quantitative PCR (qPCR) was performed using the *ND1* primers (forward 5′-AGTCACCCTAGCCATCATTCTACT-3′ and reverse 5′-GGAGTAATCAGAGGTGTTCTTGTGT-3′) and *COX3* primers (forward 5′-AGGCATCACCCCGCTAAATC-3′ and reverse 5′-GGTGAGCTCAGGTGATTGATACTC-3′), obtained from Thermo Fisher Scientific, and 20 ng template DNA/sample. The PCR conditions were as follows: 95 °C for 10 min, 40 cycles of 95 °C for 15 s and 60 °C for 60 s.

mtDNA copy numbers were corrected to the nuclear control gene RNAse-P (nDNA; cat. no. 4403326; Thermo Fisher Scientific) using human genomic DNA for the standard curve. mtDNA copy numbers were calculated using Copy Caller software (Thermo Fisher Scientific) and expressed as a mtDNA/nDNA ratio^11^.

### Renal injury marker

KIM-1 levels were measured using enzyme-linked immunosorbent assays with a commercial kit (Cat. No. MBS264966; MyBioSource, San Diego, CA, USA) according to the manufacturer’s protocol and standardized with urine creatinine levels (ng/mg), as previously described^46^. We measured KIM-1 levels in the IgAN group and HVs at the time of kidney biopsy and in the IgAN group at 6 months after medical treatment. A single investigator performed all measurements blinded to the clinical information.

### Structural differences in IgAN compared with normal kidneys

We examed structural changes in the mitochondria in two patients with IgAN using transmission electron microscopy. A kidney biopsy specimen from a living kidney donor who had normal kidney function was assessed as a control. To detect mitochondrial ultrastructure morphology, the kidney samples were cut into 1 mm^3^ pieces using a scalpel. Ultrathin sections were stained with uranyl acetate and lead citrate, and examined using a digital electron microscope (JEM-1400; JEOL USA, Peabody, MA, USA). All glomeruli were examined at x3000 and examined at a higher magnification for mitochondria structure^47^.

### Statistical analysis

Descriptive characteristics of the study population were reported as means ± standard deviations or medians with interquartile ranges for continuous variables and as frequency counts with percentages for categorical and binary variables. Comparisons of differences between groups were made using Mann-Whitney and Wilcoxon signed rank tests for continuous variables and either χ^2^ tests or Fisher’s exact tests for categorical variables, as appropriate. Urinary mtDNA copy numbers were log-transformed. Spearman’s rank correlation coefficient was used to analyze the relationships between urinary mtDNA copy numbers and clinical and histological variables. All statistical tests were two-sided, and the results were presented with 95% confidence intervals. We considered *p* values of less than 0.05 to indicate statistical significance. All analyses were performed using SPSS 25 for Windows (SPSS Inc., Chicago, IL, USA).

## Data Availability

The datasets generated during and/or analyzed during the current study are available from the corresponding author on reasonable request.
